# The Impact of Childhood Abuse and Neglect on the Development of Features of Polycystic Ovary Syndrome: A Pilot Study

**DOI:** 10.1089/whr.2024.0130

**Published:** 2025-04-10

**Authors:** Akanksha Misra, Olivia Wolfe, Ricardo Azziz

**Affiliations:** ^1^ SUNY Plattsburgh, Plattsburgh, New York, USA.; ^2^ Depts. of Obstetrics & Gynecology, Heersink School of Medicine, University of Alabama at Birmingham (UAB), Birmingham, Alabama, USA.; ^3^ Depts. of Medicine, Heersink School of Medicine, UAB, Birmingham, Alabama, USA.; ^4^ Dept. of Healthcare Organization & Policy, School of Public Health, UAB, Birmingham, Alabama, USA.; ^5^ Dept. of Health Policy, Management and Behavior, School of Public Health, University at Albany, SUNY, Albany, New York, USA.

**Keywords:** PCOS, menstrual dysfunction, adverse childhood experience, emotional abuse, childhood neglect

## Abstract

**Objective:**

To determine associations of childhood emotional and physical abuse and neglect with the incidence of menstrual irregularity, male pattern hair growth, and possible PCOS.

**Design:**

Cross-sectional Study at University.

**Subjects:**

410 individuals, 18–45 years old.

**Intervention:**

Survey.

**Main Outcome Measure(s):**

A questionnaire was administered to students, faculty, and staff at a regional State University of New York (SUNY) campus. Data on sociodemographic factors, menstrual dysfunction (irregularity, male pattern hair growth, and PCOS), and experiences of childhood abuse and neglect were collected.

**Results:**

Participants were sub-grouped into those with menstrual irregularity (MI: defined as >35 days between one period’s beginning and the next, or ≤8 cycles/year, or absent periods altogether), or male pattern hair growth (MHG: defined as excess hair on the upper lip, chin, chest, abdomen, buttocks, or back), both (MI+MHG), or those who did have neither (Unaffected). Family income status yielded some association with the presence of MI, MHG, or MI+MHG. There were significant correlations between individuals reporting MI, MHG, and MI+MHG and reported experiences of feeling loved by their caregiver (*p* value = 0.0029988), experiencing verbal abuse (*p* value = 0.0000004293), experiencing physical neglect (*p* value = 0.030228), feeling emotionally disconnected from their caregiver (*p* value = 0.0006138), and not having a peaceful home (*p* value = 0.00005760630462), vis-à-vis Unaffected individuals. Almost all respondents with a prior diagnosis of PCOS (97.6%) reported MI and/or MHG.

**Conclusions:**

All childhood experiences of abuse and neglect, except the loss of a parent, were significantly associated with features suggestive of PCOS. Larger, unbiased population studies across different demographics, are needed.

## Introduction

Polycystic ovary syndrome (PCOS) is known to affect 8–14% of the reproductive age population^1^ yet studies examining social determinants of health (SDoH) remain scarce, with a few exceptions,^2,3^ especially in the context of the United States. In recent years, there has also been growing discussion around Adverse Childhood Experiences (ACEs) in public health and policy work and its limitations.^4,5^ ACEs in reproductive and maternal health are increasingly being studied, sometimes in tandem with intermediary determinants such as perceived life stressors, for various conditions such as early onset of menarche, gestational age, fetal outcomes, psychosocial issues during pregnancy, and chronic physical and mental health conditions more generally, to name a few, with higher number of ACEs associated with higher incidence of these conditions.^6–9^ ACEs may lead to dysregulated maturation of the hypothalamic–pituitary–ovarian axis and/or the development of metabolic dysfunction, *e.g.,* insulin resistance, which would serve as precursors for menstrual and reproductive dysfunction, including PCOS.^10,11^

There are also recent studies on how specific kinds of ACEs are associated with the development of common features of PCOS, such as menstrual irregularity and unwanted excess hair growth in a male-like pattern (*i.e.,* hirsutism), and might also co-exist with a higher number of psychiatric disorders.^12,13^ Both these studies have in particular highlighted the impact of emotional abuse in childhood and ACE score of ≥4 as the strongest predictor of psychological disorders as compared to other lifestyle factors and stressors. However, there continues to be a discrepancy in the way ACE categories are defined and used in studies. While most studies have gone with the original ACE questions (or combinations thereof), a recent scoping review suggested that there seem to be inconsistencies in how ACEs are defined.^14^ Perhaps more importantly, the same study and others have indicated the limitations of ACE applicability because operationalization of ACE results usually links ACE scores with health outcomes (downstream solutions) instead of focusing on structural/societal and preventative measures from the bottom up that create those outcomes to begin with.^14–17^ These studies have recommended expanded ACEs to include factors like experiencing racism with minoritized populations and evaluating ACEs in the context of local geographies and SDoH to implement more upstream or structural changes to fix long-term health inequities by linking ACEs to structural issues.^18^

Based on these studies, we hypothesized that exposure to ACEs, especially emotional abuse and neglect, along with structural factors or SDoH like race, education, and income levels, particularly in a rural area where studies have shown a higher incidence of ACEs,^19^ will increase the risk of ovulatory dysfunction, excess male-like hair growth, and possibly PCOS. To begin to assess the impact of ACEs, specifically emotional and physical abuse and neglect and their interaction with SDoH factors of race, education, and income on the development of PCOS symptoms and possible diagnosis, we carried out a pilot study, undertaking a college-wide survey of 18–45-year-old rural college population. We now report on the results of our pilot survey.

## Materials and Methods

### Study subjects

The SUNY regional campus of our study is located in North Country, New York, a part of the largely rural and agricultural Adirondack North Country region. It is an undergraduate comprehensive college with a total enrollment of 4474 students in Fall 2022 (including graduate students), with 2778 female-identifying students. As of Spring 2023, there are 881 employees (faculty and staff) on the state payroll, of which 485 identify as female. The college is a PWI (Primarily White Institution), with approximately 64% and 83% of its student population at the undergraduate and graduate levels, respectively, identifying as non-Hispanic white in the 2021–2022 academic year. Nearly half of the college students also identify as first-generation, drawing mainly from weak socioeconomic backgrounds in the North Country region and New York City.

The North Country of New York State is primarily a rural region and, in general, is one of the most economically depressed regions of the state, with household median incomes for the entire region well below the state median.^20^ The region also suffers from a persistent lack of healthcare facilities and providers, which has been compounded by the Covid pandemic. For example, this is highlighted by the high “Health Professional Shortage Areas” score assigned to North Country counties like Clinton and Franklin.^21^ Unsurprisingly, across all indicators of child well-being and child welfare, as of 2022, Clinton and Franklin counties in North country also score persistently low compared to the state average.^22,23^

### Survey tool

We conducted a campus-wide survey open to all post-menarchal students, faculty, and staff, irrespective of gender identity in the college, between March and May of 2023 (see Supplementary Data). The following six childhood neglect parameters were assessed: (i) feeling unloved/unwanted as a child, (ii) verbal abuse as a child (*e.g.,* being sworn at, insulted, or being put down) by a caregiver, (iii) feeling emotional disconnect from caregiver despite having physical needs met as a child, (iv) not having physical needs met as a child (*e.g.,* lack of food or shelter, wearing dirty clothes, and not having anyone to protect them), (v) the loss of a parent (*e.g.,* through divorce, abandonment, death, and other), and (vi) lack of a peaceful home (*e.g.,* where adult caregivers were constantly Fighting, had drug addictions, were depressed or mentally unwell, or went to prison). Our questions regarding childhood neglect were adapted from the typically used standard ACE questionnaires.^24,25^ Questions mostly corresponded to emotional abuse and neglect (questions (i), (ii)**, **(iii), physical neglect (iv), and general household dysfunction as reflected in questions (v) and (vi)). In addition, the survey included questions regarding demographics, menstrual function, excess facial and body hair growth, prior diagnosis of PCOS, and use of hormonal therapy.

For the two variables of feeling unloved and feeling an emotional disconnect from the caregiver despite having physical needs met (i and iii), we intentionally introduced an additional categorical response, in addition to “yes” and “no,” called “it’s complicated.” Questions about emotions such as feeling neglected/unloved or not having a sentimental bond with your parents/caregivers can be potentially triggering as well as being hard to answer in a simple yes/no format unless individuals have already thought through them, which might be difficult given the young age of the overwhelming number of our research participants. Moreover, applied research and critique of ACEs have shown that although useful, standard ACE questions may not necessarily capture the breadth of lived experiences of people,^26^ questions like “feeling loved” especially may be subject to “individual interpretation,”^27^ females have more complex patterns vis-à-vis male of childhood adversity,^28^ and the lack of structural context of the study.^4^

However, the addition of the subjective, categorical “it’s complicated” response does limit the quantitative replicability of the survey tool for these two variables vis-à-vis a continuous, quantifiable, Likert-scale response.

The survey was monitored in two modalities- online *via* Google form and in paper format (see Supplementary Data). It was initially piloted with 10 participants for readability and accessibility before being widely circulated on campus during the spring semester of 2023 (March–May). Most surveys were handed out to instructors for distribution in class (depending on their preferred modality) and some were directly administered by us in different classrooms. Surveys were also distributed *via* QR codes shared with prominent university offices and in locations such as the Student Health Center, the Office of Diversity, Equity, and Inclusion, and so on. The survey was also widely circulated in employee and student digests for the duration of the semester. Faculty and staff also took the survey as it was also widely distributed across administrative offices, including janitorial staff and other administrative personnel. Participants were not remunerated and were asked to leave their email addresses only if they desired follow-up. We obtained approval for this study from the college IRB Ethics Committee (#1739). All participants were provided informed consent as a part of the survey completion. No funds were utilized for the survey.

### Outcome variables

We included the following outcomes in the study: the presence of Menstrual Irregularity (MI), presence of Male-pattern hair growth (MHG), presence of both MI and MHG (MI+MHG) and Unaffected (presence of neither MI nor MHG). Participants with menstrual irregularity (MI) were defined as those with infrequent or irregular cycles that were either more than every 35 days between the beginning of one period and the next, or 8 or fewer cycles per year, or altogether absent periods. The presence of excessive “male like” hair growth (MHG) was defined by a self-reported male-like hair growth either on the upper lip, chin, chest, abdomen, buttocks or back. Participants reporting both MI and MHG were grouped as MI+MHG and the remaining population was grouped as Unaffected.

### Statistical analyses

All participants were sub-grouped into MI, MHG, MI+MHG, and Unaffected categories. Since the survey relied on self-reporting of PCOS, and PCOS is often under-diagnosed and reported,^29^ we not only explicitly asked if people had been diagnosed with PCOS and how (doctor’s diagnosis, self-diagnosis, using apps, through family/friends, or ‘other’), we also checked for self-reported menstrual irregularity and hirsutism, since their presence or absence seems to be a good predictor for PCOS.^30^ Therefore, we grouped participants into these 4 combinations of MI and MHG (or absence thereof) instead of simply the presence or absence of PCOS as an outcome, to capture more potential PCOS numbers that might otherwise not get included, due to our inability to diagnostically test for PCOS as a part of a college survey. All analyses were conducted using R programming using standard chi-square tests for independence with *p* value set for standard confidence interval of 95% and statistically significant *p* value falling below 0.05. However, due to multiple contingent variables, *p* values were further adjusted using Bonferroni correction to avoid false positive results.

## Results

We received 554 responses in total. After review, we further eliminated duplicate and empty surveys, surveys with people who reported never having menstruated (question 9, Supplementary Data), those who were over the age of 45, and those self-reporting peri/menopause-related menstrual irregularities. Duplicate surveys were identified in a handful of participants who had left their emails and filled out exactly the same answers in two surveys, so we eliminated one copy of the two. (Tables 1 and 2).

**Table 1.** Sociodemographic Variables and Dysfunction

	Breakdown by reported MI and MHG					Adjusted *p* value
Categories (%)	MI *n* = 104 (25.4%)	MHG *n* = 76 (18.5%)	MI+MHG *n* = 96 (23.4%)	Unaffected *n* = 134 (32.7%)	Raw *p* value	Bonferroni
Race						
White	67 (64.4)	43 (56.6)	53 (55.2)	91 (67.9)		
Black	11 (10.6)	12 (15.8)	11 (11.5)	23 (17.2)	0.1374	0.687
Latinx	9 (8.6)	5 (6.6)	11 (11.5)	5 (3.7)		
Others (Asian, Indigenous, Bi, Multi-)	17 (16.3)	16 (21.1)	21 (21.9)	15 (11.2)		
Current income status						
None	18 (17.3)	5 (6.6)	10 (10.4)	16 (11.9)		
Less than $35K	17 (16.3)	23 (30.3)	28 (29.2)	21 (15.7)	0.0099	0.0495
$35K–74,999K	27 (26)	16 (21.1)	24 (25)	37 (27.6)		
$75K–99,999K	11 (10.6)	13 (17.1)	9 (9.4)	26 (19.4)		
>$100K	30 (28.8)	13 (17.1)	21 (21.9)	32 (23.9)		
Undeclared	1 (1)	7.9	4 (4.2)	2 (1.5)		
Highest education of caregiver						
Less than high school	6 (5.8)	2 (2.6)	5 (5.2)	7 (5.2)		
High school	30 (28.8)	26 (34.2)	29 (30.2)	39 (29.1)	0.9215	
University	67 (64.4)	47 (61.8)	62 (64.6)	88 (65.7)		1
Undeclared	1 (1)	1 (1.3)	0 (0)	0 (0)		
PCOS present?						
Yes	17 (16.3)	11 (14.5)	52 (54.2)	2 (1.5)		
Don’t know what that means	22 (21.2)	17 (22.4)	19 (19.8)	24 (17.9)	<2.2e-16	1.100e-15
No	65 (62.5)	48 (63.2)	25 (26)	108 (80.6)		
Hormonal or any medications in past 3 months						
Yes	65 (62.5)	39 (51.3)	56 (58.3)	50 (37.3)	0.0004998	0.002499
No	39 (37.5)	37 (48.7)	40 (41.7)	82 (61.2)		
Undeclared	0 (0)	0 (0)	0 (0)	2 (1.5)		

MHG, male pattern hair growth; MI, menstrual irregularity; PCOS, polycystic ovary syndrome.

**Table 2.** Childhood Neglect and Dysfunction

	Breakdown by reported MI and MHG					Adjusted *p* value
Categories (%)	MI *n* = 104 (25.4%)	MHG *n* = 76 (18.5%)	MI+MHG *n* = 96 (23.4%)	Unaffected *n* = 134 (32.7%)	Raw *p* value	Bonferroni
Feeling loved vs. unwanted						
Felt loved	65 (62.5)	44 (57.9)	38 (39.6)	103 (76.9)	0.0004998	0.0029988
It’s complicated	33 (31.7)	28 (36.8)	47 (49)	27 (20.1)		
Clearly felt unloved	6 (5.8)	4 (5.3)	11 (11.5)	4 (3)		
Verbal abuse						
Yes	33 (31.7)	26 (34.2)	55 (57.3)	26 (19.4)	0.00000007155	0.0000004293
No	71 (68.3)	50 (65.8)	41 (42.7)	108 (80.6)		
Emotional disconnect from caregiver						
Yes	47 (45.2)	45 (59.2)	58 (60.4)	62 (46.3)	0.0001023	0.0006138
It’s complicated	25 (24)	12 (15.8)	21 (21.9)	12 (9)		
No	32 (30.8)	19 (25)	17 (17.7)	60 (44.8)		
Physical negligence						
Yes	10 (9.6)	9 (11.8)	16 (16.7)	4 (3)	0.005038	0.030228
No	94 (90.4)	67 (88.2)	80 (83.3)	130 (97)		
Loss of parent (multiple reasons)						
Yes	29 (27.9)	26 (34.2)	36 (37.5)	33 (24.6)	0.155	0.93
No	75 (72.1)	50 (65.8)	60 (62.5)	101 (75.4)		
Peaceful home						
Yes	60 (57.7)	38 (50)	34 (35.4)	92 (68.7)	9.601e-06	0.000057606
No	44 (42.3)	38 (50)	62 (64.6)	42 (31.3)		

The 410 remaining surveys were included in the analysis. Of included respondents, 94.4% identified as female, 1.2% as male, and 4.4% as ‘other’ gender; 61.9% identified as White, 13.9% as Black, 7.3% as Latinx, and 16.8% as ‘Other’ including Indigenous, Asian, and Bi-or Multi-racial; 87.8% were 18–25 years of age, 3.7% were 26–35 years of age, and 8.5% were 36–45 years of age. As expected, 88.3% of respondents were students, 6.3% were staff, and 5.4% were faculty. Of all respondents, 26.1% had been raised in the North Country and 58.1% outside of the North Country but within New York State. Only 7.8% reported being raised outside of the state and 8.1% outside of the country.

Respondents were then sub-grouped based on the reported presence of menstrual irregularity (MI) (self-reported as “Yes” or “No”), the reported presence of excessive “male like” hair growth (MHG) (self-reported as “Yes” or “Some”), or the presence of both (MI+MHG). We found that 25.4% of our population reported MI but no MHG, 18.5% MHG but no MI, 23.4% both MI and MHG, and 32.7% were Unaffected.

Due to multiple contingent variables, all *p* values were adjusted using Bonferroni correction.

Table 1 presents the results of five variables, including three SDoH variables: respondents’ race, current family income level (self-income if they are faculty or staff and caregiver income if they are students), highest education attainment of their primary caregiver, prior diagnosis of PCOS, and use of hormonal medications vs. MI, MHG, MI+MHG, and Unaffected. Neither race nor the highest level of primary caregiver education demonstrated a significant association with the presence of MI and/or MHG. With family income level, the adjusted *p* value was just under 0.05 (0.0495), indicating association. The presence of a prior diagnosis of PCOS and the use of hormonal medications in the prior 3 months strongly correlated with the presence of these features.

Table 2 presents the results of the six childhood experiences of neglect variables assessed vs. MI, MHG, MI+MHG, and Unaffected. All six parameters of childhood neglect studied, except the loss of a parent, demonstrated a significant association with the presence of MI, MHG, or MI+MHG. However, given the lack of appropriate psychometrics to capture variables of feeling unloved and feeling emotional disconnect from the caregiver despite having physical needs met (i and iii) in which we introduced a new categorical variable “it’s complicated,” we report our results as preliminary and needing further investigation in conjunction with revisions to the survey measurement and our ongoing qualitative research (see section Discussion).

## Discussion

Our pilot study suggests that experiences of childhood neglect, except the loss of a parent, are significantly associated with features suggestive of possible PCOS, including menstrual dysfunction and unwanted male-like hair growth. Notably, respondents who reported *both* MI and MHG, the group with 97.6% of respondents with a prior diagnosis of PCOS, also reported the highest proportion of childhood neglect. These data are in agreement with the study by Tay and colleagues assessing psychiatric comorbidities and ACEs in women with self-reported PCOS in a population-based survey study of Australian women^13^ and Pringle and colleagues assessing the association of childhood maltreatment with PCOS in a case-control study of psychiatrically healthy South African women ages 18–79.^12^

Tay et al. derived their results from a large longitudinal dataset of a representative sample population of Australian women born between 1989 and 1995. They found that self-reported PCOS, along with other lifestyle factors, ACEs, and sociodemographic variables (age, education level, employment status, and relationship status) were correlated with reported psychiatric disorders. When taken together in a multivariable analysis, these factors were attenuated but mostly remained significantly associated with psychiatric disorders, but the most significant factors were ACEs (≥4). Pringle et al. derived their results from mixed ancestry populations of female control subjects of a cross-sectional study in Cape Town, South Africa. Of the sociodemographic factors they evaluated, level of education and marital status were associated with PCOS but not on multivariate analyses. Emotional and physical abuse were significantly associated with PCOS, but only emotional abuse remained significant when other types of child maltreatment were accounted for. In our present study as well, SDoH like race and education levels of childhood caregivers were not associated with the presence of features suggestive of PCOS. One reason for race and education levels not showing association in our sample might be that the student population at the college, who made up 88.3% of the 410 participants included, largely comes from low socioeconomic status (SES) households, irrespective of race. In addition, the vast majority of students at the college are first-generation. In support, almost 33.7% reported a household income of either none or less than $35,000 per year. As in a previous study, we reported an association between low SES and PCOS,^2^ the data regarding family income status showed some association, with the adjusted *p* value = 0.0495. Moreover, as the same study has indicated, PCOS seems to be more prevalent in women with low SES and higher education levels, raising the question of whether it is the SES or the higher awareness of PCOS that comes with higher education levels that are impacting the prevalence of PCOS. Since this is a college population, it may be the combination of low SES and education levels of the subjects that are leading to higher self-reporting of MI, MHG, and MI+MHG. Moreover, since family income levels for faculty and staff may not be identical to their family income levels growing up, with many of the local staff and faculty coming from low SES backgrounds, the association between SES and MI, MHG, MI+MHG might be stronger than indicated in our survey.

The data for emotional (verbal) abuse and lack of a peaceful home environment, which have significant overlap, demonstrates a particularly significant association with menstrual dysfunction and/or excess male-like hair growth, and possibly PCOS. This is consistent with the findings of Pringle and colleagues that emotional abuse remains significantly associated with PCOS even after factoring in all subtypes of abuses and neglect.^12^

The lack of association between loss of parent due to death, divorce, abandonment, imprisonment, or any other reason and PCOS related features might once again be representative of our student population, who primarily come from low SES, first generation, and non-traditional, single parent backgrounds. Also, loss of parent doesn’t necessarily lead to loss of a peaceful home and/or emotional and physical abuse and neglect. Other studies have shown associations between inflammatory biomarkers and age of menarche and menopause with childhood adversities like parental loss,^31,32^ but quality studies looking specifically at parental loss, endocrine-metabolic functioning, and impacts on reproductive health and associated disorders such as PCOS and other psychiatric conditions, remain scarce. One study in Sweden on ante- and postnatal depression showed correlation between all standard ACEs except loss of parent with depression.^33^ Therefore, parental loss might be working in combination with other ACEs and sociodemographic factors like poverty to increase risk for menstrual dysfunction or irregularities as in PCOS.^34^

The strengths of this study include the relatively large number of respondents, the fact that respondents were in the optimum age range for PCOS-associated features, and the use of a validated childhood neglect assessment tool. Furthermore, our data suggests that the questions used to identify individuals at high risk for PCOS were valid. The fact that the prevalence of prior diagnosis of PCOS was strongly associated with menstrual dysfunction and/or male-like excess hair growth suggests that these survey questions did identify a group of individuals at greater risk of PCOS. These data are consistent with our prior study demonstrating that the responses to these questions (regarding menstrual dysfunction and male-like excess hair growth) have a high negative and positive predictive value for PCOS.^30^ Also consistent with this assumption, it is not surprising that those individuals with menstrual dysfunction and/or excess male-like hair growth also demonstrated the highest proportions of using hormonal therapy in the preceding three months.

Our pilot study does have significant limitations, principal of which is the fact that we did not proceed to clinically verify the presence of PCOS in those individuals reporting features of menstrual dysfunction and/or excess male-like hair growth. Nonetheless, as noted above, we previously reported a high predictive value for the responses to these questions and the presence of PCOS verified clinically. Furthermore, given that 67.3% of the 410 respondents included in our analysis reported menstrual dysfunction and/or excess male-like hair growth, it is likely that there was significant self-selection bias among respondents.

Another significant limitation in our study is the introduction of the new categorical variable “it’s complicated” for assessing emotional abuse and neglect variables of feeling unloved and feeling emotional disconnect from the caregiver despite having physical needs met. We are currently awaiting analysis of our now almost complete qualitative data from semi-structured interviews of volunteer participants, most of which have a formal diagnosis of PCOS, and local histories of the North Country region, which help ground the survey data in local context. These interviews have revealed to us certain prominent themes in childhood experiences, which when juxtaposed with the survey data can reveal a clearer picture of the correlation between emotional and physical abuse and negligence and PCOS. This is also enabling us to unpack the categorical variable of “it’s complicated” in a deeper and more context-specific way, even more than asking people to grade their emotional responses on a continuous, Likert scale, which we recommend using in the future in the questionnaire. While Likert responses improve data reliability,^35^ they can also be equally inadequate when it comes to context-specific emotional abuse or neglect in childhood, which co-occurs with multiple adversities,^34^ something we are hoping our qualitative data to reveal. Similarly, while there is critique that the ACE questionnaire could be subjected to recall bias, research has shown both prospective and retrospective ACE measures to be associated with later adult health outcomes and has shown to have good reliability.^36^ The high number of respondents answering “yes” or “It’s complicated” for both these questions, especially for individuals reporting MI, MHG, or MI+MHG gives us a preliminary indication that there might be a connection between emotional experiences of neglect and menstrual dysfunction and/or PCOS.

Also, our questions regarding childhood neglect focused mainly on emotional and physical abuse and neglect and not childhood sexual abuse. We did not include question(s) on sexual abuse because we did not want to compromise an already compromised young and mostly low-income college going rural population. Also, studies are showing that when it comes to menstrual outcomes like PCOS specifically, emotional adverse experiences may be more significant than sexual abuse, even though childhood sexual abuse could cause other conditions such as early age of menarche.^12,32^ We also did not include the question about realization of abuse in later life (question 23, see Supplementary Data) in our analysis because we felt that that was not a measure of the experience of neglect during childhood.

We also recognize the limitation of our sample, drawn from a rural college going population in Northeastern New York. While the correlations between ACEs and PCOS therefore cannot be immediately generalized to larger populations, we employ a more expansive notion of generalizability, drawing on innovative social science research methodologies that are place-specific and are also being increasingly picked up by scientific studies.^37,38^ Our local, rural, community-oriented research draws on Fine’s conceptualization of generalizability as both theoretical and provocative generalizability, which go beyond population samples to ask how problems move from one context to another under uneven global topographies and urge us into action.^39^ So for example, while our data is not showing correlation of PCOS with some sociodemographic factors such as race and education due to the specificities of our sample because of the specificities of our population or because SDoH play out in combined and more intricate ways,^40^ it is showing a strong association with emotional abuse, which has been demonstrated in recent, reputable studies^12,13^ across different geographies. This should move us to action to study how ACEs like emotional abuse (and in our study also neglect), which are so hard to capture, work with other SDoH grounded in particular locations to increase risk for menstrual dysfunction or irregularities as in PCOS.

In summary, results of our pilot survey suggest that there appears to be an association between emotional abuse and emotional and physical neglect with the presence of menstrual dysfunction and/or excess male-like hair growth, features highly predictive of PCOS. How childhood abuse and neglect results in PCOS is unclear, although early childhood stress, as mentioned earlier, may lead to dysregulated maturation of the hypothalamic–pituitary ovarian axis and/or the development of metabolic dysfunction, *e.g.,* insulin resistance, which would serve as precursors for PCOS. Further studies, including larger, unbiased populations across different demographic locations, are needed to examine the impact of emotional and physical abuse and neglect in childhood on the development of menstrual dysfunction, excess male-like hair growth, and PCOS. More qualitative data, including in-depth interviews with people diagnosed with PCOS, along with better quantitative survey instruments, are also needed to capture nuances and memories of childhood that are often complicated and difficult to recall. Some high-quality qualitative studies on PCOS exist,^41,42^ but these are usually in larger urban and global contexts, not in rural regions of America where, as we have stated above, endemic healthcare shortage and high rates of poverty and childhood abuse remain persistent. All of these results, including ours, indicate not only the significance of ACEs on development of PCOS and menstrual health in general, but also the impossibility of not looking at ACEs as or in conjunction with other SDoH. While ACEs show a strong correlation in our study to possible PCOS over other SDoH like race, education and to some extent SES, it is important to look at these variables together, especially in rural areas where poverty and child welfare, race and gender inequities co-exist^40^ to create stressors in early life impacting menstrual function.

## Data Availability

Data regarding any of the subjects in the study has not been previously published unless specified, and the data will be made available to the editors of the journal for review or query upon request. The data underlying this article will be shared upon reasonable request to the corresponding author.

## Abbreviations Used

ACE: Adverse Childhood Experience
MHG: Male pattern hair growth
MI: Menstrual Irregularity
PCOS: Polycystic ovary syndrome
SDoH: Social determinants of health
