# Supplementary material for: The Impact of Childhood Abuse and Neglect on the Development of Features of Polycystic Ovary Syndrome: A Pilot Study
Source: Womens Health Rep (New Rochelle). 2025 Apr 10;6(1):412–20. doi: 10.1089/whr.2024.0130 (PMC12040527; doi:10.1089/whr.2024.0130)
Supplement: Supplementary Data [file whr.2024.0130_supplementary_data.pdf]

PCOS and Childhood Experiences: Initial Screening Questionnaire

Questions marked with an asterisk * are mandatory

1. What best defines your role at SUNY Plattsburgh?*
   - ☐ Student
   - ☐ Faculty
   - ☐ Staff
   - ☐ Other

2. What is your age?*
   - ☐ 18-25 years
   - ☐ 26-35 years
   - ☐ 36-45 years
   - ☐ Over 45 years

3. What race do you identify with?*
   - ☐ White or Caucasian
   - ☐ Black or African American
   - ☐ Latinx or Hispanic
   - ☐ Asian (including South, East and Southeast Asia)
   - ☐ Native or Indigenous American
   - ☐ Native Hawaiian or Other Pacific Islander
   - ☐ Bi/Multi Racial

4. What gender do you identify with?*
   - ☐ Female
   - ☐ Male
   - ☐ Other

5. Where were you born?*
   - ☐ In or around Plattsburgh or the North Country
   - ☐ In New York State but not in the North Country
   - ☐ In another state
   - ☐ Outside of the US

6. Where were you primarily raised?*
   - ☐ In or around Plattsburgh or the North Country
   - ☐ In New York State but not in the North Country
   - ☐ In another state
   - ☐ Outside of the US

7. If faculty or staff, what is your annual gross income? If student, please check the box that applies for whoever financially supports you (self, parents, extended family, etc.)*
   - ☐ None
   - ☐ Less than $34,999
   - ☐ $35,000-$74,999
   - ☐ $75,000-$99,999
   - ☐ $100,000-$199,999
   - ☐ Greater than $200,000

8. What is the highest degree that any one of your parents or the person who raised you had?*
   - ☐ Less than high school
   - ☐ High School graduate
   - ☐ Bachelors
   - ☐ Masters
   - ☐ Ph.D.

9. Have you ever menstruated (gotten periods)?*
   - ☐ Yes
   - ☐ No (skip to question 17)

10. Do you have Polycystic Ovarian Syndrome (PCOS), to the best of your knowledge?
   - ☐ Yes
   - ☐ Don't know what that means/Unsure (skip to question 13)
   - ☐ No (skip to question 13)

11. How do you know you have PCOS?
   - ☐ Doctor's diagnosis
   - ☐ I figured it out myself
   - ☐ Friends/Family told me
   - ☐ I took an online test and/or used an app
   - ☐ Other

12. How long have you been diagnosed with PCOS?
   - ☐ Less than 1 year
   - ☐ 1-5 years
   - ☐ 6-10 years
   - ☐ Over 10 years

13. Are you on any hormonal medications (birth control pills, hormone medications, etc.) or have used any other medications in the past 3 months?
    - ☐ Yes
    - ☐ No

14. Do you have infrequent or irregular cycles; either more than every 35 days between the beginning of one period and the next, or 8 or less cycles per year OR absent periods?
    - ☐ Yes
    - ☐ No (skip to question 16)

15. Do you have any one of the following conditions contributing to irregular or absent periods:
    - ☐ Perimenopause/Menopause
    - ☐ Birth control medication
    - ☐ Surgery
    - ☐ Another underlying physical or mental health issue for which I have gone to the doctor
    - ☐ Another underlying physical or mental health issue for which I have NOT gone to the doctor
    - ☐ Pregnancy/Postpartum/Breastfeeding
    - ☐ None of the above

16. Do you have "male-like" hair growth on your upper lip, chin, chest, abdomen, buttocks, or back?
    - ☐ Yes
    - ☐ No
    - ☐ Some. Not sure how "excessive" it is

17. Growing up, did your parents or caregiver make you feel loved and special? Or did you have a sense of feeling unwanted, a "burden"?*
    - ☐ Yes, my parent/caregiver made me feel loved and special
    - ☐ No, I constantly felt unwanted
    - ☐ It's complicated

18. Growing up, did a parent or adult in your home **regularly** swear at you, insult you, or put you down?*
    - ☐ Yes
    - ☐ No

19. Growing up, did you ever have all your physical needs met (food, shelter, clothes, education...) but still feel an emotional disconnect from your parents or any other adult caregivers at home?*
    - ☐ Yes
    - ☐ No
    - ☐ It's complicated

20. Growing up, did you feel that you didn't have enough to eat, had to wear dirty clothes, or had no one to protect or take care of you?*
    - ☐ Yes
    - ☐ No

21. Growing up, did you lose a parent through divorce, abandonment, death, or any other reason?*
    - ☐ Yes, at least one of those reasons
    - ☐ No

22. Growing up, did you live in a "peaceful" environment or were your parents or adult caregivers at home constantly fighting and/or had drug addictions and/or were depressed or mentally unwell and/or went to prison?*
    - ☐ It was a peaceful home with none of the above problems
    - ☐ No. I lived with at least one of the problems mentioned above

23. Have there been moments in your life now or in the past years, as an adult, having left your home environment, when you have realized you may have been emotionally or physically neglected, betrayed, not nourished well (good food and also emotionally) by the people who were supposed to raise you?*
    - ☐ Yes, but I haven't done anything about it
    - ☐ Yes, and I have sought mental help through therapy, etc.
    - ☐ No

You're done!

Thank you so much for taking this survey. If you don't mind, please leave your email address, in case you can be contacted for a very brief follow-up survey or interview in which you will be compensated for your time with a gift card!

Email: ____
